# Efficacy and tolerability of vildagliptin as first line treatment in patients with diabetes type 2 in an outpatient setting

**DOI:** 10.1186/s40200-015-0194-6

**Published:** 2015-08-22

**Authors:** M. P. Yavropoulou, M. Pikilidou, K. Kotsa, A. Michopoulos, E. Papakonstantinou, J. G. Yovos

**Affiliations:** Division of Endocrinology and Metabolism, 1st Department of Internal Medicine, AHEPA University Hospital, Aristotle University of Thessaloniki, Thessaloniki, Greece; 1st Department of Internal Medicine, AHEPA University Hospital, Aristotle University of Thessaloniki, Thessaloniki, Greece; Laboratory of Clinical and Molecular Endocrinology, 1st Department of Internal Medicine, AHEPA University Hospital, 1 S. Kyriakidi street 54636, Thessaloniki, Greece

**Keywords:** First line treatment of diabetes type 2, Vildagliptin, DPP-4 inhibitors

## Abstract

**Background:**

Inhibitors of dipeptidyl-peptidase IV are recommended as second-line therapy in type 2 diabetes (DT2), but data, as a first-line treatment in everyday clinical practice are scarce. To address this issue we conducted a 12-month, clinical study in an outpatient setting, using vildagliptin as the first-line treatment.

**Methods:**

Ninety-one drug naïve patients with DT2 started with vildagliptin monotherapy (100 mg daily) for 4 months and were scheduled to regular 4-monthly visits for 1 year. Patients received add-on treatment with metformin or metformin and glimepiride according to their glycosylated hemoglobin (HbA1c) at each study-visit.

**Results:**

HbA1c was significantly decreased with vildagliptin monotherapy from 8.16 % ± 1.60 to 7.52 % ± 1.60, *p* < 0.001. Only 39 % of the patients achieved the target of HbA1c ≤ 7.0 % at the end of the 4th month. Mean change in HbA1c was significantly correlated with baseline HbA1c values (r = −0.51, *p* < 0.001). At the end of the study only 35 % of the patients remained on vildagliptin monotherapy while the rest required add-on treatment with metformin or metformin and sulfonylurea.

**Conclusions:**

Vildagliptin is well tolerated either as monotherapy or in combination but the majority of patients require add-on therapy shortly after the beginning of treatment.

## Background

Drugs focusing on the incretin system, such as dipeptidyl peptidase 4 inhibitors (DPP-4i) and glucagon–like peptide −1 (GLP-1) analogues have been recently introduced in the clinical practice for the treatment of diabetes type 2 demonstrating efficacy and a favorable safety profile [1]. Orally administered DPP-4i increase circulating concentrations of endogenous active GLP-1, although to a lesser degree compared to GLP-1 analogues [2] and lower glucose levels by stimulating insulin secretion and inhibiting glucagon secretion.

In the recent consensus for the treatment of diabetes [3] DPP-4i, such as vildagliptin and sitagliptin are recommended as second-line therapy in combination with either metformin or sulfonylureas. In patients with diabetes type 2 who do not reach the glycemic targets with metformin alone, DPP-4 inhibitors as add-on treatment can efficiently lower glycosylated hemoglobin (HbA1c), with neutral effects on body weight. Compared with sulfonylureas or thiazolidinediones, DPP-4 inhibitors exert similar hypoglycemic efficacy and in addition are associated with a lower incidence of hypoglycemia or other serious adverse events.

As first line therapy, DPP-4 inhibitors can be an alternative therapeutic option in patients who cannot tolerate metformin because of gastrointestinal adverse events, and several clinical studies have proved their efficacy as monotherapy in drug-naïve patients with diabetes type 2. However, data are scarce regarding DPP-4 inhibitors as the first choice for diabetes management in everyday clinical practice.

To address this issue we conducted a clinical study in the outpatient setting using vildagliptin, which is a potent and selective DPP-4i, as the first line treatment in drug –naïve patients with newly diagnosed diabetes type 2.

## Methods

This was a 12-month clinical study that was conducted in the outpatient setting of the Diabetes center of the Endocrinology Division. All patients with newly diagnosed diabetes type 2 according to WHO criteria that visited the outpatient clinic were initially screened for eligibility. Exclusion criteria were secondary diabetes, prior treatment with oral hypoglycemic agents or insulin, malignancy, acute diabetic complications, myocardial infarction, unstable angina, or coronary artery bypass surgery within the previous 6 months, congestive heart failure, NYHA Class III or IV, liver disease and renal insufficiency (CrCl < 45 ml/min).

All participants provided written informed consent. The protocol was approved by the ethics committee of AHEPA University Hospital and the study was conducted in accordance with the Declaration of Helsinki.

### Study design

**Fig. 1:**
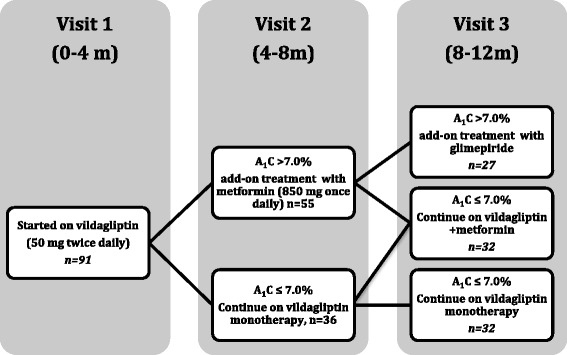
Flow chart of the study design. Ninety one patients were enrolled in the study. After 4 months of vildagliptin monotherapy 36 patients reach the target of HbA1c ≤ 7.0 % and continue on vildagliptin treatment, while 55 patients needed add-on treatment with metformin. At the end of the 8th month of treatment and according to HbA1c levels 27 patients needed triple therapy with glimepiride, 32 patients were on vildagliptin and metformin and 32 patients continue on vildagliptn monotherapy

Eligible patients were initially left with lifestyle modification instructions (diet and exercise) for 1–3 months without any other particular treatment. Patients that failed to achieve acceptable glucose levels (ie. FBG < 1120 and PBG < 200) after this period were finally enrolled and scheduled on regular visits every 4 months for one year. At visit 1 all patients received monotherapy with vildagliptin (50 mg twice daily) for 4 months. At the end of the fourth month period (visit 2), subjects that were not adequately controlled according to their HbA1c (<7.0 %), received add-on therapy with metformin (850 mg once daily). During the 3rd visit (at the end of the 8th month) patients that did not reach the target of HbA1c <7.0 % with metformin plus vildagliptin received add-on therapy with a sulfonylurea (Glimepiride 2 or 3 mg twice daily) (Fig. 1). All patients received a BMI adjusted diabetic diet designed by a specialized dietician of the diabetes center in the outpatient clinic that included 50–55 % of carbohydrates, 30 % fat and 15–20 % f proteins. The calculation of the daily kcals was performed by a relevant application (Bmapp, iOS Developer program, USA) that uses the BMI and the age of the patient and calculates the ideal weight. Adjustments at each visit were based on changes of the body weight of the patient. Patients who in visit 1 had triglycerides levels above 200 mg/dl and/or total cholesterol > 240 mg/dl started treatment with atorvastatin 20 mg.

According to the treatment applied during the study patients were classified into 3 groups. Group 1: Received vildagliptin for the whole study period (12 months). Group 2. Received vildagliptin for 4 and vildagliptin plus metformin for the remainder 8 months, Group 3: Received vildagliptin for 4 months, vildagliptin plus metformin for 4 months, and vildagliptin plus metformin plus sulfonylurea for the remainder 4 months (Fig. 1). Glycosylated hemoglobin, fasting (FBG) and postprandial blood glucose (PBG), body weight, fasting lipid profiles (triglycerides; TG, total cholesterol; TC, LDL, HDL) standard hematology and biochemistry laboratory assessments and vital signs were measured at each study visit. The PBG at each visit was self-assessed the previous day 2 h after their regular meal by the glucose-meter provided by the outpatient clinic.

Blood pressure (BP) measurements were performed at each study visit in the morning with the subjects in the seated position following a 5 min quiet resting period. Blood pressure was measured in both arms with a mercury sphygmomanometer using an appropriately sized cuff. Values for systolic BP (SBP) and diastolic BP (DBP) were defined by Korotkoff phase I and IV respectively. All patients were provided with glucose monitoring devices and supplies and instructed on their use during their first visit. Hypoglycemia was defined as self-monitored blood glucose below 50 mg/dl with or without symptomatology suggestive of low blood glucose. Adverse events (AEs) were recorded at each study visit and were assessed as to severity using the criteria used by the ICH Guideline for Clinical Safety Data Management (http://www.ema.europa.eu). If an adverse event results in death is life-threatening,- requires inpatient hospitalization or prolongation of existing hospitalization, − results in persistent or significant disability/incapacity, or leads to discontinuation of the treatment is categorized as severe. Classification of mild or moderate adverse events and potential relationship to the anti-diabetic treatment were based on the physicians’ opinion taken into consideration the duration of the event and the patient’s report.

### Assays

All laboratory assessments were made using standard techniques in the Central Laboratory of AHEPA University hospital. HbA1c was measured with an ion exchange HPLC method.

### Statistical analysis

The primary outcome was change from baseline in HbA1c. Secondary outcomes included fasting and postprandial glucose levels, fasting plasma lipids, and body weight. All values are presented as mean ± standard deviation (SD), or standard error of the mean (SE) when stated. We used analysis of covariance (ANCOVA) to test whether baseline values of HbA1c were significant in determining the change of HbA1c (Δ HbA1c), after administration of vildagliptin.

Changes from baseline in primary and secondary endpoints were analyzed using an analysis of variance (ANOVA). Pearson correlation coefficient (r) was used to test the relationships between mean changes of glucose parameters after treatment. The trapezoidal rule was used to determine the area under the curve (AUC).

We also used repeated measures analysis to determine whether treatment with vildagliptin was effective over time. Linear regression analysis was performed to create an algorithm that quantifies the net effect of vildagliptin therapy at various values of baseline HbA1c. A *P* value < 0.05 was considered statistically significant. Data were analyzed using SPSS 16.0 (SPSS Inc., Chicago, IL, USA).

## Results

Ninety-one patients aged between 39 and 84 years old (mean age 68 years) were finally enrolled in the study. Anthropometric characteristics and baseline laboratory values are summarized in (Table 1). Concomitant diseases included arterial hypertension (*n* = 15), thyroid disease (*n* = 48), osteoporosis and other metabolic bone diseases (*n* = 13), hypogonadism (*n* = 6) and hyperlipidemia (*n* = 2). Patients with previously diagnosed hyperlipidemia were on treatment with atorvastatin 20 mg/day for approximately 2 years (*n* = 2) or rosumvastatin 5 mg/day for 3,5 year (*n* = 1) and during laboratory examinations their lipid profile was within normal values. However, at visit one 31 patients had triglycerides >200 mg/dl and 25 of them had also total cholesterol >240 mg/dl. We prescribed atorvastatin 20 mg/d in these patients. Concomitant medications (including statins) remained unchanged during the study period. Family history of diabetes was positive in 71 % of the patient population. Blood pressure measurements were within normal values during the study period.

**Table 1:** Baseline and anthropometric characteristics of the study population

N	91
Age (yrs)	68.4 ± 11.3
Μen (%)	55 (60 %)
BMI (kg/cm^2^)	28 ± 5.7
Obese patients (n,%)	28,30 %
Fasting blood glucose (mmol/l)	10.3 ± 2.4
HbA_1_c (%)	8.16 ± 1.6
Postprandial blood glucose (mmol/l)	11.7 ± 3.4
Urea (mmol/l)	10.67 ± 2.7
Creatinine (umol/l)	79.56 ± 26.52
Total Cholesterol (mmol/l)	5.7 ± 1
HDL (mmol/l)	1.15 ± 0.3
LDL (mmol/l)	3.6 ± 1.0
Triglycerides (mmol/l)	2.17 ± 1.0

### 4 months monotherapy with vildagliptin

Glycosylated hemoglobin was significantly decreased after 4 months monotherapy with vildagliptin from 8.16 % ± 1.60 at baseline to 7.52 % ± 1.60, *p* < 0.001. The mean change of HbA1c was −0.6 and 39 % of the patients (*n* = 36) achieved the target of HbA1c ≤ 7.0 % at the end of the 4th month. Similarly, significant reductions were observed in FBG, PBG and serum triglycerides level (FBG: 185.5 ± 43.2 mg/dl vs. 135 ± 23.4 mg/dl, *p* < 0.001, PBG: 210.8 ± 61.2 mg/dl vs. 165.7 ± 28.8 mg/dl *p* < 0.001, TG: 186 ± 88.5 vs. 141.7 ± 62 mg/dl, *p* < 0.001). Total cholesterol levels were also reduced, albeit not significantly, after 4 months of treatment with vildagliptin (220.4 ± 38.6 mg/dl vs. 208.8 ± 34.8 mg/dl *p* = 0.0719), while there was no significant change in body weight. Changes in HbA1c were unrelated to changes in FBG and PBG levels.

Patients with baseline HbA1c values ≤ 8.0 % achieved the target of HbA1c <7.0 % to a significantly greater percentage, compared to patients that had baseline values above 8 % (46 % vs. 25 %, *p* < 0.001, respectively). Mean change in HbA1c was significantly and inversely correlated with baseline HbA1c values (r = −0.51, *p* < 0.001), and this relationship remained robust after adjusting for changes in body weight (ΔBMI), age, gender and baseline FBG and PBG.

A linear regression analysis was used to create a statistical model that quantifies the effect of HbA1C at baseline on post-treatment HbA1c and concluded to the following algorithm:

*Δ*HbA1c = 2, 40–0, 41 × HbA1c_baseline_, which also remained significant (R^2^ = 29,8 %, *p* < 0,001), after adjusting for ΔBMI, age, gender and baseline FBG and PBG.

Mean change in fasting glucose levels (50.9 mg/dl) was significantly and inversely correlated with baseline values of fasting glucose (r = −0.848, *p* < 0.001). Mean change in postprandial glucose levels (37.9 mg/dl) was also significantly correlated with baseline fasting (r = −0.269, *p* = 0.029) and postprandial glucose levels (r = −0.882, *p* < 0.001). There was no significant change in body weight.

### 12 month comparative study with vildagliptin monotherapy versus combination therapy

During visits 2 and 3 patients either remained on vildagliptin monotherapy (*n* = 32) or received add-on treatment with metformin (*n* = 32) or received add-on treatment with metformin and glimepiride (*n* = 27) according to whether the HbA1c target of below 7.0 % was achieved (Fig. 1).

Repeated measurements analysis for group 1 that received vildagliptin monotherapy during the whole study period, confirmed that there was no difference on the effect of vildagliptin overtime and that was true for both males and females. There was no significant difference between baseline values of fasting and postprandial glucose and fasting lipids between the 3 groups, except for HbA1c, as expected by study design (Table 2). Similarly there were no significant between-groups differences in mean changes in FBG and PBG (Table 3). However, when we used AUC for the assessment of fluctuation of HbA1c at the study intervals, AUC _HBA1C 1–12_ was significantly higher in group 3 compared to groups 2 and 1 (6.27 ± 0.66 % vs. 5.54 ± 0.43 % vs. 4.7 ± 0.44, *p* < 0.001, respectively). This was not the case when assessing AUC for FBG (AUC _FBG1–12_) and PBG (AUC _PBG1–12_).

**Table 2:** Baseline values of diabetic patients treated with vildagliptin monotherapy versus ccombination treatment

Baseline values	Group 1 (Vildagliptin)	Group 2 (Vildagliptin +metformin)	Group 3 (Vildagliptin+metformin +glimepiride)	*P*
N	32	32	27	–
Age (yrs)	59.7 ± 10.2	63.1 ± 12.3	65 ± 10.5	0.23
Μen (%)	20 (62 %)	22 (68 %)	13 (48 %)	–
BMI (kg/cm^2^)	28 ± 5	27 ± 4.6	29.6 ± 7.5	0.34
Fasting blood glucose (mmol/l)	10.5 ± 2.9	10.4 ± 2.1	9.7 ± 1.9	0.64
HbA1c (%)	7.4 ± 1.3	8.1 ± 1.6	9 ± 1.3	<0.001
Postprandial glucose (mmol/l)	11.2 ± 3.6	11.65 ± 2.9	12 ± 3.7	0.23
Total Cholesterol (mmol/l)	6.1 ± 0.9	5.6 ± 1.2	5.8 ± 0.8	0.46
HDL (mmol/l)	1.1 ± 0.2	1.2 ± 0.3	1.1 ± 0.3	0.30
LDL (mmol/l)	3.7 ± 0.9	3.3 ± 1.1	3.6 ± 0.8	0.41
Triglycerides (mmol/l)	1.9 ± 0.6	2.3 ± 1.3	2.3 ± 0.9	0.37
Systolic Blood pressure (mmHg)	120.9 ± 8.3	118.9 ± 7.2	121 ± 8.4	0.33
Diastolic Blood pressure (mmHg)	80.6 ± 7.3	79.9 ± 6.8	89.6 ± 7.3	0.47

**Table 3:** Mean changes in glucose and lipids before and after treatment for 12 months

Mean change of tested parameters	Group 1	Group 2	Group 3
Μean ± SE	Vildagliptin monotherapy	Vildagliptin +Metformin	Vildagliptin+ Metformin +Glimepiride
Mean change in HbA1C (%)	0.9 ± 0.22 % *p* < 0.001	1.2 ± 0.27 % *p* < 0.001	1.4 ± 0.25 % *p* < 0.001
Mean change FBG (mg/dl)	68.57 ± 8.5 mg/dl *p* < 0.001	55.17 ± 9.6 mg/dl *p* < 0.001	101 ± 14 mg/dl *p* < 0.001
Mean change PBG (mg/dl)	−37.24 ± 10 mg/dl *p* < 0.005	56.4 ± 13 mg/dl *p* < 0.001	120.2 ± 24.2 *p* < 0.001
Mean change triglycerides (mg/dl)	24.7 ± 16 mg/dl *p* < 0.001	52 ± 21 mg/dl *p* = 0.01	71 ± 33 mg/dl *p* = 0.27
Mean change total cholesterol (mg/dl)	12 ± 2 mg/dl *p* = 0.32	13 ± 10 mg/dl *p* = 0.21	17 ± 9 mg/dl *p* = 0.06
Mean change HDL (mg/dl) ^a^	6.47 ± 4 mg/dl *p* = 0.02	4 ± 3 mg/dl *p* = 36	10 ± 5 mg/dl *p* = 0.26
Mean change LDL (mg/dl)	20.4 ± 6 mg/dl *p* = 0.77	41 ± 18 mg/dl *p* = 0.93	50 ± 13 mg/dl *p* = 0.08
Mean change BW (kg)	0.2 0.2 ± 0.03 *p* = 0.32	1.2 ± 0.2 1.2 *p* = 0.4	1.3 ± 0.6 kg 1.3 *p* = 0.48

^a^HDL level was increased after treatment

At the end of study period 90 % of the patients in the vildagliptin monotherapy group, 56 % in the vildagliptin plus metformin group and 44 % of the vildagliptin plus metformin plus sulfonylurea group achieved the target of HbA1c <7.0 %.

### Mean changes of the parameters tested at the end of the study

Mean changes in glucose levels, HbA1c, lipids and weight are shown in Table 3. The mean change ± SE in HbA1c from baseline to end point was larger in group 3 (−1.4 ± 0.25 %), compared to group 2 (−1.2 ± 0.27 %) and group 1 (−0.9 ± 0.22 %), albeit not significantly (Table 3).

There was a significant reduction in FBG and PBG at the end of the study in all groups. Triglycerides and total cholesterol levels were significantly reduced in group 1 and group 2 while significant changes were seen in HDL and LDL levels in groups 1 and 3, respectively.

### Adverse events

No confirmed symptomatic or asymptomatic hypoglycemia was reported during the study period. Most of the AEs that were reported were mild or moderate in severity, did not lead to discontinuation of the anti-diabetic treatment and included nausea (*n* = 12) and gastrointestinal discomfort (*n* = 5). One patient in the vildagliptin monotherapy group reported an oedema in the lower extremities, which was resolved in the first month of treatment.

In addition no adverse reactions attributed to drug- interactions were reported during the study period. No major changes from baseline to endpoint were observed for any of the routinely assessed hematological or biochemical parameters.

## Discussion

The efficacy and safety of vildagliptin as monotherapy have been widely confirmed in a large body of clinical studies in various populations with type 2 diabetes [4–8]. In our study and in agreement with previous data, vildagliptin monotherapy (100 mg daily) produced a clinically significant decrease in HbA1c as a first-line treatment in drug-naïve patients with type 2 diabetes. However, the reduction of HbA1c observed was lower in the older age patients probably due to the longer duration of diabetes in this group and was also lower compared to randomized clinical trials, probably due to the nature of our study design that do not allow us a tight control on the patient’s diet and compliance to study drugs, which were both self-reported.

Moreover we have shown a favorable lipid profile in all three groups of treatment with significant reductions in serum triglycerides and LDL cholesterol and significant increases in HDL in combination with atorvastatin.

A small number of patients, achieved the target of 7.0 % in the first 4 months of vildagliptine monotherapy (36 %) but most of them (approximately 90 %) sustain HbA1c <7.0 % at least for 1 year after the first diagnosis of their diabetes, in line with previous results demonstrating a sustained effect of vildagliptin over time [8]. At the end of study period 90 % of the patients in the vildagliptin monotherapy group, 56 % in the vildagliptin plus metformin group and 44 % of the vildagliptin plus metformin plus sulfonylurea group achieved the target of HbA1c <7.0 %. Based on the algorithm that was developed by data from our patients we found that the main determinant of vildagliptin efficacy as monotherapy in drug-naïve patients was, baseline HbA1c as it has also been shown for vildagliptin plus metformin [9]. In a recent study by Takeshita et al., when vildagliptin was compared to liraglutide as a second line treatment after sitagliptin baseline levels of docosahexanoic acid but not eicosapentanoic acid demonstrated a significant predictive value for vildagliptin- mediated improvement in glycemic control independent of its effects on insulin secretion and insulin sensitivity [10]. Detailed lipid parameters were not measured in our study. In addition, our patients were drug naïve when vildagliptine was initiated and thus our results are not directly comparable with the study by Takeshita et al. [10].

Vildagliptin was well tolerated either as monotherapy or in combination with metformin or metformin and glimepiride, and no episodes of hypoglycemia were observed.

Currently, most type 2 diabetes patients begin and continue with the gold standard treatment of metformin [11]. When metformin is not tolerated due to gastrointestinal side effects, such as diarrhea and nausea [12] or is contraindicated, as in congestive heart failure and renal disease, alternative first line treatments include sulfonylureas, thiazolidinediones and DPP-4i. Compared to the first two drug categories DPP-4i are equally efficacious in lowering HbA1c and have a favorable safety profile with low risk of hypoglycemia due to the incretin-based mechanism of action and neutral effect on body weight.

In studies comparing vildagliptin to metformin vildagliptin has proven non-inferiority and was better tolerated with fewer gastrointestinal adverse events [13–15].

Clinically relevant efficacy of vildagliptin was also seen compared to rosiglitazone [16–18]. Both drugs decreased HbA1c to a similar extent at short term with vildagliptin showing some weight benefit [16]. Extension of this study [16] over 2-years in drug-naïve diabetic patients also demonstrated statistically significant and sustained HbA1c reductions for both drugs with rosiglitazone showing greater durability in lowering HbA1c at the expense of significant weight gain and less favorable plasma lipid profile compared with vildagliptin [17].

Compared to gliclazide, vildagliptin produced similar HbA1c reductions over 2-years monotherapy but non-inferiority was not established [19] despite the benefits of vildagliptin in weight and hypoglycemia. In patients with low baseline values of HbA1c vildagliptin and glimepiride as add on treatment to metformin demonstrated similar efficacy in reducing HbA1c with markedly reduced hypoglycemia risk and no weight gain in the vildagliptin plus metformin treated group [20, 21].

Overall, monotherapy with vildagliptin or vildagliptin plus metformin controlled adequately HbA1c levels in 73 % of drug-naïve patients with newly diagnosed diabetes after 12 months of treatment in line with previous results [4, 9, 22–24]. Patients that needed add-on treatment with sulfonylurea were those with more pronounced postprandial hyperglycemia and mean baseline values of HbA1c around 9 %. We did not find significant differences between treatment groups from baseline to end point in the glucose parameters tested, probably due to the small sample size in each group.

## Conclusions

In summary we have shown that monotherapy of vildagliptin as first line treatment or in combination with metformin in the outpatient setting is an effective approach in a considerable number of patients albeit with an increased cost relative to more conventional alternatives. Additional benefits such as low risk of hypoglycemia, neutral effect on body weight and favorable lipid profile can be particularly appreciable in selective patients with low baseline HbA1c levels or in the elderly population, as has been recently shown [25].
